# Eosinophilic Pneumonia Induced by Daptomycin

**DOI:** 10.7759/cureus.55095

**Published:** 2024-02-27

**Authors:** Juan D Ayala Torres, Brian Noreña, Alma Tatiana Suarez Poveda

**Affiliations:** 1 Radiology, Universidad de Antioquia, Medellín, COL

**Keywords:** eosinofilia pulmonar, daptomycin, drug-related side effects and adverse reactions, pulmonary eosinophilia, radiology

## Abstract

Daptomycin-induced eosinophilic pneumonia (DIEP) is a rare but serious complication associated with the use of this broad-spectrum antibiotic. We present the case of a teenager with a history of nasopharyngeal cancer who developed DIEP while receiving daptomycin to treat an infection associated with an implanted chamber catheter. Symptoms included recurrent dyspnea and peripheral eosinophilia, with radiological findings consistent with DIEP. The pathophysiology involves an immune response triggered by daptomycin, resulting in eosinophilic pulmonary inflammation. Diagnosis requires a thorough evaluation of medical history, clinical laboratory tests, and radiological findings. The main treatment involves discontinuation of daptomycin and, in severe cases, the use of steroids. It is essential to consider DIEP in patients with respiratory failure and bilateral pulmonary opacities who have used daptomycin and to suspect it in those with blood eosinophilia or in bronchoalveolar lavage.

## Introduction

Various lung diseases have been associated with medication toxicity, among which eosinophilic pneumonia (EP) has been described in association with the use of antibiotics, nonsteroidal anti-inflammatory drugs, cardiovascular medications, antidepressants, and anticonvulsants [1-2]. Daptomycin is an antibiotic with spectrum activity against gram-positive bacteria, usually used as a second-line treatment after vancomycin for Methicillin-resistant *Staphylococcus aureus* (MRSA) infections in skin, soft tissues, and bacteremia [3-4]. It has been linked to diseases such as acute respiratory distress syndrome (ARDS), organizing pneumonia, pleuroparenchymal fibroelastosis, and, as in this case, EP, which is a rare complication [1-5]. This article aims to present a case of daptomycin-induced eosinophilic pneumonia (DIEP) and review the characteristics of the disease.

## Case presentation

**Figure 1:**
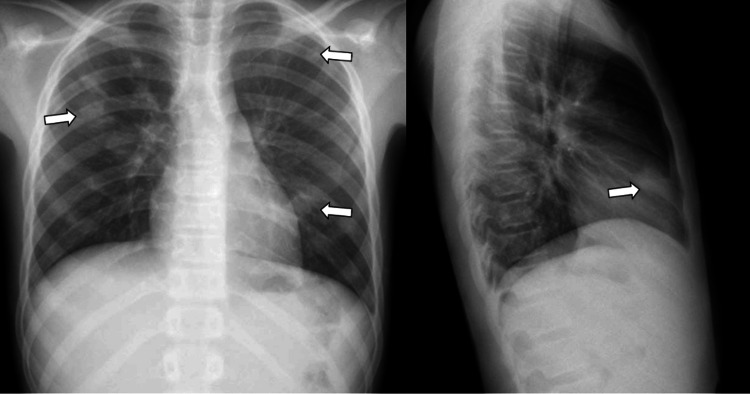
Chest X-ray in PA and lateral projection Multiple bilateral nodular opacities of variable size, some of them with small radiolucent foci within them, suggest cavitations (arrows). PA: posteroanterior

**Figure 2:**
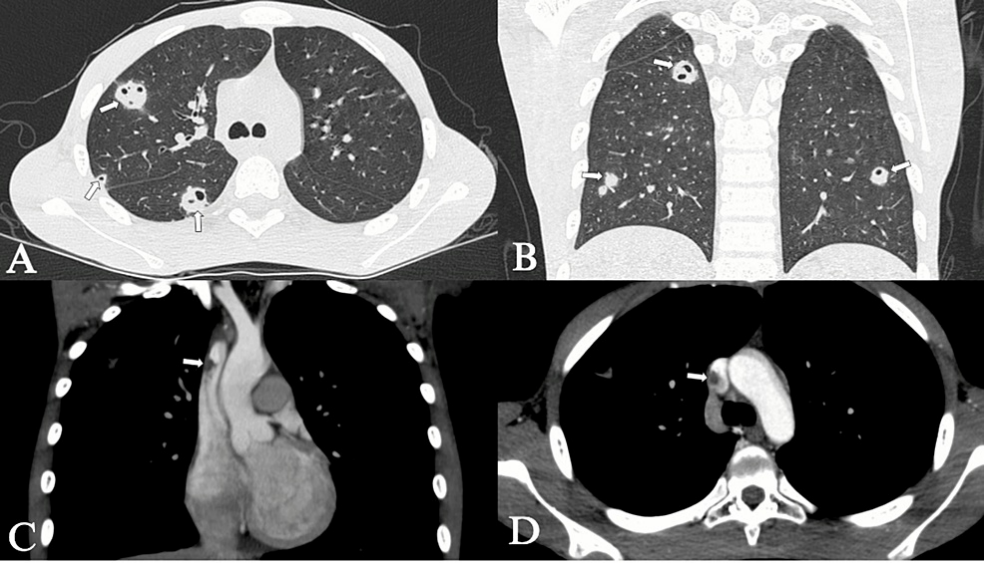
Chest CT Chest CT in the simple lung parenchyma window in the axial plane (A) and coronal plane (B) and contrast-enhanced chest CT in the soft tissue window in the coronal plane (C) and axial plane (D). Bilateral peripheral solid nodules of variable size, some of them cavitated (arrows in A and B), correlating with the opacities seen on the chest X-ray. Partial thrombosis of the superior vena cava (arrows in C and D). CT: computed tomography

We report the case of a 15-year-old adolescent with a history of undifferentiated nasopharyngeal carcinoma, stage T4 N1 M0, treated with surgical resection, chemotherapy with cisplatin, and radiotherapy. During his hospital stay, he developed an infection associated with the implanted chemotherapy port catheter and bacteremia complicated by MRSA with pulmonary nodules, some cavitated, secondary to septic emboli, and superior vena cava thrombosis identified on conventional radiography (Figure 1) and CT of the chest (Figure 2).

**Figure 3:**
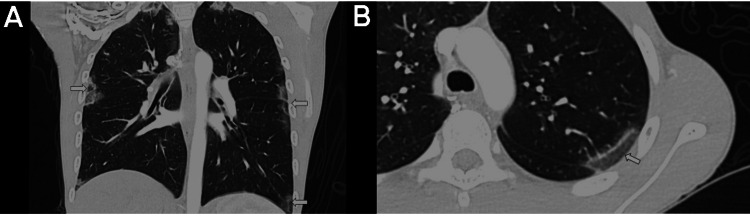
Chest CT with intravenous contrast in the lung parenchyma window Coronal plane (A) and axial plane (b). Bilateral peripheral ground-glass opacities (arrows) involving all lung lobes associated with the thickening of interlobular septa; some of the opacities are surrounded by a denser consolidation border, forming the atoll sign or reverse halo (Arrow in B). CT: computed tomography

**Figure 4:**
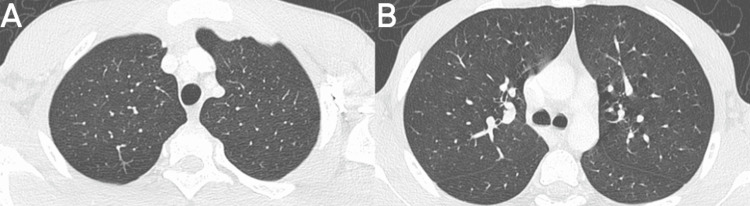
High-resolution chest CT two months after discontinuation of daptomycin in the axial plane and lung parenchyma window Resolution of parenchymal opacities present in previous studies (A and B). CT: computed tomography

Initially, he received treatment with vancomycin for one week without clinical improvement. The infectious diseases department recommended switching to daptomycin with a regimen of 400 mg orally daily for seven days. One week after completing the antibiotic treatment, the patient experienced mild shortness of breath, cough, and eosinophilia (4400/µl). A CT of the chest identified bilateral peripheral ground-glass opacities, some with the reverse halo sign (Figure 3). After reviewing the association with daptomycin use, the diagnosis of DIEP was made. Due to the mild symptoms, corticosteroid treatment is not considered. At the pulmonology follow-up two months after hospital discharge, the patient is asymptomatic and has resolved the findings of the chest CT (Figure 4).

## Discussion

EPs are characterized by eosinophilic tissue inflammation and peripheral eosinophilia. Previously, a minimum duration of six months was considered (persistent eosinophilia), but currently, it is not deemed necessary due to delays in diagnosis and treatment. [6]. EPs are categorized into those of unknown causes: (1) simple pulmonary eosinophilia, (2) acute eosinophilic pneumonia (AEP), (3) chronic eosinophilic pneumonia (CEP), (4) idiopathic hypereosinophilic syndromes, and (5) eosinophilic bronchitis; and those of known causes: (1) allergic bronchopulmonary aspergillosis (ABPA), (2) bronchocentric granulomatosis, (3) those associated with parasitic and fungal infections, (4) those secondary to radiotherapy or medications, and (5) eosinophilia associated with vasculitis in eosinophilic granulomatosis with polyangiitis (EGPA) [2,7].

The diagnosis of DIEP requires an evaluation of medical history, laboratory tests, and imaging findings [3,8]. The pathophysiology remains unclear; it is believed that daptomycin irreversibly binds to surfactant and acts as an antigen that is phagocytosed by alveolar macrophages, leading to an inflammatory cascade and tissue damage upon presenting the antigen to TH2 lymphocytes, which release inflammatory factors such as interleukin 5 and eotaxin, resulting in eosinophil production and recruitment to inflamed sites [3-4].

Clinical symptoms include skin rashes, fever, respiratory symptoms such as dry cough, progressive dyspnea requiring oxygen supplementation, and ARDS [7,9]. Peripheral eosinophilia is defined as an absolute eosinophil count greater than 0.5 x 109/L, while the term hypereosinophilia corresponds to two tests at least one month apart with eosinophils in blood greater than 1.5 x 109/L. Its importance as a diagnostic test lies in guiding and ruling out other conditions with similar presentations, leading to timely treatment [10]. Alveolar eosinophilia in bronchoalveolar lavage (BAL) is determined when the differential count exceeds 10-25%, with normal being less than 1% [7-8]. Immunoglobulin E (IgE), erythrocyte sedimentation rate (ESR), and C-reactive protein (CRP) are also often elevated [3,7-8]. A pulmonary biopsy may be required to confirm the diagnosis [3].

Imaging findings are nonspecific and may present patterns of simple pulmonary eosinophilia, AEP, CEP, or EGPA [7-8,11]. The chest X-ray shows bilateral alveolar and interstitial opacities with peripheral distribution. CT of the chest reveals bilateral consolidations, nodules, and ground-glass opacities predominantly in the upper and peripheral regions, pleural effusion, and lymphadenopathy [3,7,11]. The reverse pulmonary edema sign is rare [9].

Utilizing a multifaceted approach, the disease can be effectively classified based on clinical presentation, concurrent daptomycin use, laboratory parameters, and bronchoalveolar lavage results, yielding enhanced diagnostic precision. It is considered definitive in patients with concurrent use of daptomycin, fever, dyspnea requiring oxygen or mechanical ventilation, new opacities on chest X-ray or CT, eosinophilia >25% in BAL, and clinical improvement upon antibiotic discontinuation. It is probable in patients with eosinophilia <25% in BAL or peripheral eosinophilia and other criteria of definitive disease [4]. In a possible disease, pulmonary opacities improve with daptomycin discontinuation without eosinophilia [4]. Treatment involves discontinuation of daptomycin and steroids in severe cases [7,9,11].

Other pathologies can also cause eosinophilia, including organizing pneumonia, idiopathic pulmonary fibrosis, Langerhans cell histiocytosis, neoplasms, vasculitis such as EGPA, infections such as coccidioidomycosis, *Pneumocystis jirovecii*, and mycobacteria, and autoimmune diseases [2,7]. Differential diagnoses in imaging include infections, organizing pneumonia, alveolar hemorrhage, sarcoidosis, ARDS, and neoplasms [8,11].

## Conclusions

DIEP should be considered in the diagnosis of patients with respiratory failure and bilateral pulmonary opacities who are using or have recently used daptomycin and who present with peripheral eosinophilia or eosinophilia in BAL. High clinical suspicion is required for its diagnosis.
